# Celastrol attenuates diabetic kidney disease progression by repressing senescence of renal tubular epithelial cells

**DOI:** 10.3389/fragi.2025.1657947

**Published:** 2025-11-27

**Authors:** Yajun Zhang, Zewei Sun, Weixue Meng, Siqi Lei, Jingyong Sun, Yixuan Tang, Xiaodong Mu

**Affiliations:** School of Pharmaceutical Sciences, Shandong First Medical University & Shandong Academy of Medical Sciences, Jinan, China

**Keywords:** diabetic kidney disease, tripterygium wilfordii, celastrol, cell senescence, glucose

## Abstract

**Background:**

Recent investigations across both animal models and human cohorts increasingly highlight cellular senescence as a critical pathological process driving the development and progression of diabetic nephropathy (DN). The detrimental impact of senescent cells on DN advancement stems from a range of underlying mechanisms, notably telomere attrition, compromised mitochondrial function, dysregulated autophagy, chronic inflammatory responses, altered mTOR signaling and Sirtuin activity, and the release of pro-coagulant factors. Diabetic kidney disease (DKD) is a common and serious complication in diabetic patients, closely associated with high glucose-induced defects in kidney cells. Currently the clinical treatment of DKD disease is still a challenge. Celastrol, a compound derived from Tripterygium wilfordii, has shown significant therapeutic effects on DKD, but the specific mechanisms remain unclear.

**Methods:**

We established *in vitro* and *in vivo* models of DKD using human renal tubular epithelial cells (HK-2) and Sprague-Dawley (SD) rats. The effects of celastrol on glucose-induced oxidative damage to HK-2 cells and kidney injury in DKD rats were observed. The potential mechanisms were investigated through both *in vitro* and *in vivo* experiments.

**Results:**

High glucose induced accelerated senescence of HK-2 cells *in vitro,* and celastrol reversed senescence-associated pathological changes in the cells. Celastrol reduced pro-inflammatory signaling and mitochondrial damage *in vitro* by inhibiting the phosphorylation of aging- and inflammation-related proteins NF-κB and AKT1. *In vivo,* celastrol inhibited the phosphorylation of NF-κB and AKT1 in renal tissues, effectively improving renal dysfunction and pathological changes in DKD rats, and reducing disease-related indicators.

**Conclusion:**

Celastrol may be a promising candidate drug for the treatment of DKD. It can treat DKD by reversing the imbalance of the immune-inflammatory system mediated by the AKT/NF-κB/TNF-α signaling during the progression of the disease and may also delay the progression of DKD through its anti-aging effect.

## 1 Introduction

Diabetic kidney disease (DKD) is a microvascular complication characterized by hyperglycemia, persistent proteinuria, hypertension, and impaired kidney function (Anders et al., 2018; Dai et al., 2017; Yaribeygi et al., 2019; Zhang et al., 2020). It accounts for approximately 50% of end-stage renal disease cases and is a leading cause of mortality in diabetic patients (Shen et al., 2017; Gallagher and Suckling, 2016). Current treatment strategies focus on glycemic control and blood pressure management, but these approaches are insufficient to halt disease progression (Gheith et al., 2016; Liu et al., 2016).

Since diabetic tissues and organs have extensive cellular senescence (Luo et al., 2023), the role of cellular senescence in DKD has attracted widespread attention in recent years. Cellular senescence in DKD involves multiple mechanisms, including telomere shortening, DNA damage, epigenetic modification, and mitochondrial autophagy defects (Xiong and Zhou, 2019; Liu F. et al., 2024). Previous studies have shown that DKD is highly associated with accelerated senescence of renal tubular epithelial cells, mesangial cells, podocytes, and endothelial cells (D’onofrio et al., 2016; Kitada et al., 2014). In particular, hyperglycemia can also induce macrophages to secrete senescence-associated secretory components (SASP) and promote the development of low-grade inflammation, directly inducing cellular senescence of mesangial cells (Zhang et al., 2006) and renal tubular epithelial cells (Liu et al., 2015a). During DKD, the accumulation of damaged mitochondria may be the cause of premature senescence of renal tubular epithelial cells.

Cellular senescence has emerged as a critical factor in DKD pathogenesis. Hyperglycemia-induced oxidative stress, chronic inflammation, and mitochondrial dysfunction contribute to accelerated aging of renal cells, including tubular epithelial cells, mesangial cells, podocytes, and endothelial cells (Liu F. et al., 2024). The senescence-associated secretory phenotype (SASP) and the activation of NF-κB and AKT1 pathways are key mediators of this process (Zhang et al., 2019).

Celastrol, a bioactive compound derived from Tripterygium wilfordii (a traditional Chinese medicinal plant of the Celastraceae family), has demonstrated multifaceted therapeutic properties including potent anti-inflammatory, anti-aging, and mitochondrial-protective effects across various disease models (Li et al., 2024; Chen et al., 2020; Yu et al., 2025; Liu et al., 2021). As one of five traditional medicines highlighted in《Cell》with high potential for modernization, celastrol has shown efficacy in treating inflammatory disorders such as arthritis, Crohn’s disease, and Parkinson’s disease (Kannaiyan et al., 2011; Liu et al., 2015b), while also exhibiting neuroprotective effects in acute ischemic stroke (Jiang et al., 2018). And Celastrol has good anti-cancer activity in a variety of cancer diseases (Jiang et al., 2018; Pei et al., 2025). Its mechanisms involve dual modulation of inflammatory pathways and mitochondrial homeostasis - notably through PI3K-Akt signaling pathway activation to enhance mitochondrial function in muscle cells (Geng et al., 2025) and Nur77-mediated clearance of damaged mitochondria to reduce oxidative stress (Abu and Tan, 2017; Hu et al., 2017). Building on these pleiotropic actions, this study aims to explore celastrol’s therapeutic potential in diabetic kidney disease (DKD) by specifically investigating its ability to mitigate cellular senescence and inflammation, two pivotal drivers of DKD progression. The investigation will focus on how celastrol’s unique combination of immunomodulatory, antioxidant, and mitochondrial-regenerative properties may synergistically address the complex pathophysiology of diabetic nephropathy.

Since the tissues and organs of diabetic patients existing extensive accelerated cellular senescence, this study aims to systematically evaluate the therapeutic efficacy of Tripterygium wilfordii in attenuating DKD progression via anti-senescence mechanisms (Cascão et al., 2017),delineate its renoprotective effects in preclinical DKD models and elucidate the molecular underpinnings of its action, focusing on senescence-associated signaling pathways (e.g.,p16/p21, SASP modulation) (Xu et al., 2023) and mitochondrial homeostasis,in order to provide ideas and theoretical basis for the rational clinical use of tripterygium wilfordii in DKD and related research work.

## 2 Results

### 2.1 High glucose induces accelerated cellular senescence and NF-κB activation in human renal tubular epithelial cells

**FIGURE 1:**
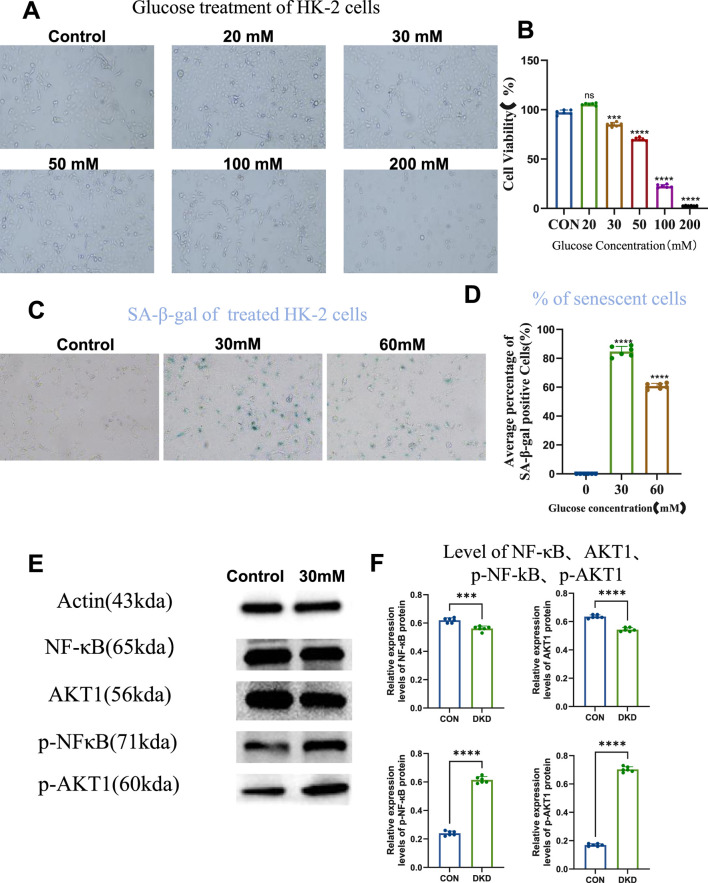
High Glucose Induces Cellular Senescence. **(A)** Cells grown under different concentrations of glucose (10×), to compare cell viability. **(B)** Statistical results of cell viability (n = 6, all data are presented as mean ± SD, ***P < 0.001 and ****P < 0.0001 compared with the control group). **(C)** SA-β-galactosidase staining to detect the level of cellular senescence (10×). **(D)** Statistical results of SA-β-galactosidase staining (n = 6, all data are presented as mean ± SD, ****P < 0.0001 compared with the control group). **(E)** Western blot analysis to detect the level of intracellular NF-кB, AKT1, p-NF-кB, and p-AKT1 proteins. **(F)** Statistical results of Western blot analysis (n = 6, all data are presented as mean ± SD, *P < 0.05, **P < 0.01, ***P < 0.001, and ****P < 0.0001 compared with the control group).

Firstly we examined whether high-glucose may induce cellular senescence of HK-2 cells, and what could be the optimal concentration. We tested the effect of different concentration of glucose on the cell viability of HK-2 cells, by studying concentrations ranging from 20 to 200 mM, and CCK-8 assay revealed that HK-2 cell viability exceeded 85% under 30 mM of glucose, establishing it as a safe concentration range (Figures 1A,B). Within this range, 30 mM and 60 mM of glucose were further tested for induction of cellular senescence. SA-β-galactosidase staining showed significantly increased percentage of senescent cell in both groups compared to controls, with 30 mM of glucose yielding the highest number of senescent cells. This confirmed 30 mM of glucose as the optimal concentration to establish an *in vitro* HK-2 cell senescence model (Figures 1C,D). Further validation using senescence-associated secretory phenotype (SASP) markers revealed elevated phosphorylated NF-κB (p-NF-κB) and AKT1 (p-AKT1) levels in the 30 mM of glucose group, despite unchanged total protein levels, indicating activation of these signaling pathways in the senescent model (Figures 1E,F). These results demonstrate successful establishment of a high-glucose-induced HK-2 cell senescence model linked to NF-κB and AKT1 phosphorylation.

### 2.2 High glucose induces increased mitochondrial damages in human renal tubular epithelial cells

**FIGURE 2:**
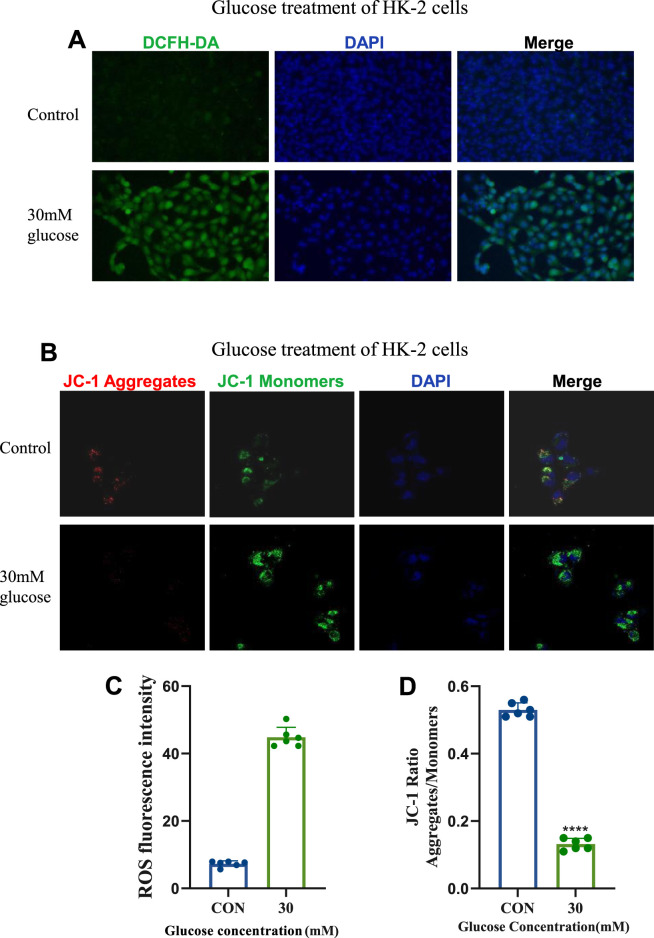
Establishment and Confirmation of the Cellular Model of Diabetic kidney disease. **(A)** ROS staining images of the control group and 30 mM glucose concentration group (20×). **(B)** JC-1 fluorescence staining images of the control group and 30 mM glucose concentration group (630×). **(C)** Statistical results of ROS staining (n = 6, all data are presented as mean ± SD, *p < 0.05, **p < 0.01, ***p < 0.001; #p < 0.05, ##p < 0.01, ###p < 0.001,compared with the control group). **(D)** Statistical results of JC - 1 fluorescence staining (n = 6, all data are presented as mean ± SD, *p < 0.05, **p < 0.01, ***p < 0.001; #p < 0.05, ##p < 0.01, ###p < 0.001,compared with the control group).

Using the DCFH-DA fluorescent probe, intracellular reactive oxygen species (ROS) were measured. DCFH-DA, non-fluorescent until oxidized by ROS to fluorescent DCF, revealed that 30 mM glucose induced significant ROS production compared to the control group, as evidenced by enhanced green fluorescence (Figures 2A,C).

Mitochondrial membrane potential was assessed using the JC-1 probe, which exhibits red fluorescence at high potential and green fluorescence at low potential. Treatment with 30 mM glucose induced mitochondrial depolarization, indicated by a shift from red to green fluorescence (Figures 2B,D), reflecting increased mitochondrial damage. These findings highlight the pathological impact of 30 mM glucose on ROS generation, and mitochondrial function in DKD-related cellular models.

### 2.3 Celastrol represses high glucose-induced mitochondrial damage and cellular senescence in human renal tubular epithelial cells

**FIGURE 3:**
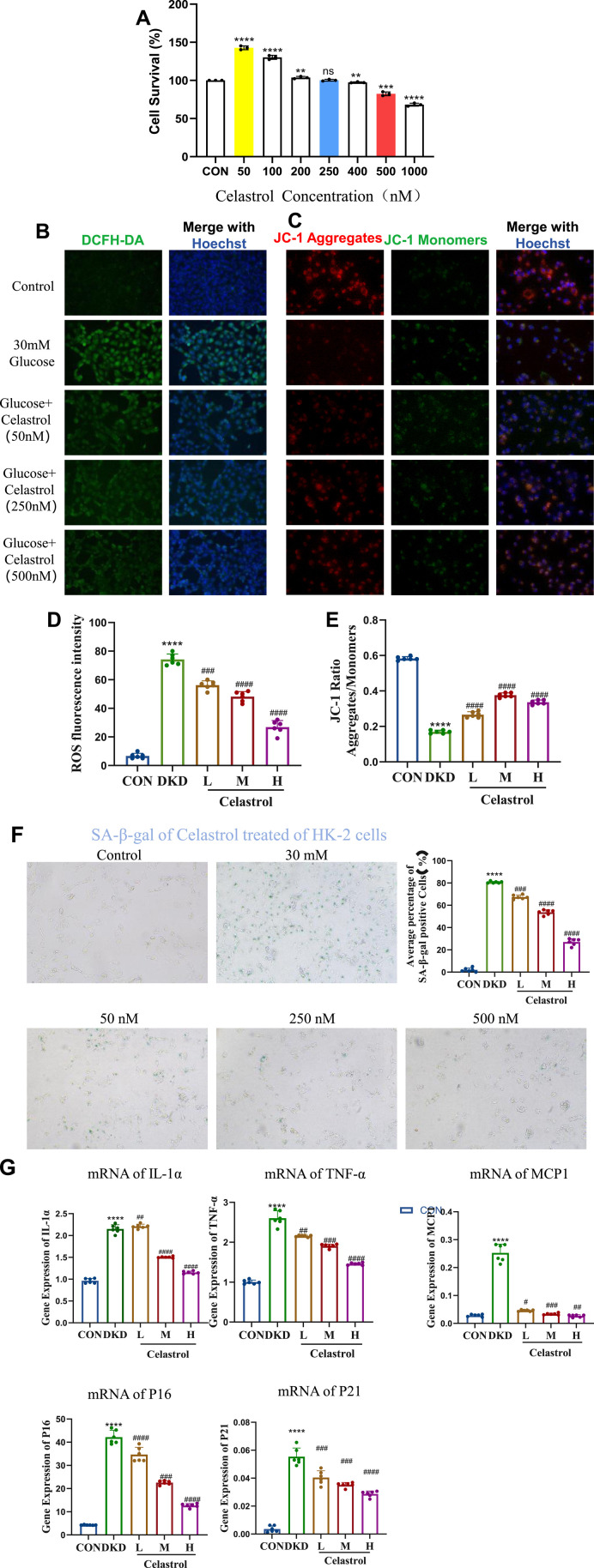
Celastrol Reduces Oxidative Stress and Mitochondrial Damage. **(A)** Statistical results of Celastrol on cell viability (n = 6, all data are presented as mean ± SD, *p < 0.05, **p < 0.01, ***p < 0.001; #p < 0.05, ##p < 0.01, ###p < 0.001, compared with the control group). **(B)** Effects of different concentrations of celastrol on intracellular ROS levels (20×) **(C)** Effects of different concentrations of celastrol on mitochondrial membrane potential depolarization (20×) **(D)** Statistical results of ROS fluorescence quantification (MET as the positive drug group; L, M, and H represent low, medium, and high concentrations, respectively. n = 6, all data are presented as mean ± SD,*p < 0.05, **p < 0.01, ***p < 0.001; #p < 0.05, ##p < 0.01, ###p < 0.001, compared with the control group; *p < 0.05, **p < 0.01, ***p < 0.001; #p < 0.05, ##p < 0.01, ###p < 0.001,compared with the DKD (30 mM) group). **(E)** Statistical results of JC-1 fluorescence quantification (L, M, and H represent low, medium, and high concentrations, respectively. n = 6, all data are presented as mean ± SD, *p < 0.05, **p < 0.01, ***p < 0.001; #p < 0.05, ##p < 0.01, ###p < 0.001,compared with the control group; *p < 0.05, **p < 0.01, ***p < 0.001; #p < 0.05, ##p < 0.01, ###p < 0.001,compared with the DKD (30 mM) group). **(F)** Effects of different concentrations of celastrol on senescent cells (n = 6, all data are presented as mean ± SD,*p < 0.05, **p < 0.01, ***p < 0.001; #p < 0.05, ##p < 0.01, ###p < 0.001,compared with the control group; *p < 0.05, **p < 0.01, ***p < 0.001; #p < 0.05, ##p < 0.01, ###p < 0.001,compared with the DKD group, i.e.,30 mM as the model group, 50, 250, 500 were the celastrol administration group). **(G)** Effects of different concentrations of celastrol on the expression of senescence-related factors (n = 6, all data are presented as mean ± SD, *p < 0.05, **p < 0.01, ***p < 0.001; #p < 0.05, ##p < 0.01, ###p < 0.001. compared with the control group; *p < 0.05, **p < 0.01, ***p < 0.001; #p < 0.05, ##p < 0.01, ###p < 0.001,compared with the DKD group).

First, we screened the administration concentration range of celastrol using the CCK-8 assay, and finally determined the low concentration (50 nM), medium concentration (250 nM), and high concentration (500 nM) (Figure 3A). Elevated reactive oxygen species (ROS) underlie metabolic and hemodynamic disruptions in diabetic kidney disease (DKD), with persistent hyperglycemia impairing mitochondrial antioxidant systems and enhancing ROS production. Here we examined celastrol’s effects on ROS and mitochondrial damage, and results showed that while 50 nmol/L celastrol had minimal impact, medium-to-high concentrations (250–500 nmol/L) significantly reduced intracellular ROS levels and mitigated mitochondrial depolarization induced by high glucose (Figures 3B-E), demonstrating a dose-dependent effect.

Regarding anti-senescence effects, SA-β-galactosidase staining revealed that 250–500 nmol/L celastrol alleviated cell senescence, though higher concentrations exerted cytotoxicity (Figure 3F). RT-qPCR analysis further indicated that celastrol suppressed high glucose-induced upregulation of senescence-associated secretory phenotype (SASP) factors (IL-1β, MCP1, TNF-α) and senescence markers (p16, p21) (Table 1) (Figure 3G). Collectively, these findings demonstrate that celastrol reduces ROS, mitigates mitochondrial damage, and exhibits anti-inflammatory and anti-aging properties in DKD models, though its precise mechanisms warrant further investigation.

**TABLE 1:** Primer sequences.

Gene	Primer sequence
GAPDH	Forward: TCCATGACAACTTTGGCATTG Reverse: TCACGCCACAGCTTTCCA
p16	Forward: GATCCAGGTGGGTAGAAGGTC Reverse: CCCCTGCAAACTTCGTCCT
p21	Forward: TGTCCGTCAGAACCCATGC Reverse: AAAGTCGAAGTTCCATCGCTC
MCP1	Forward: CAGCCAGATGCAATCAATGCC Reverse: TGGAATCCTGAACCCACTTCT
IL-1beta	Forward: ATGATGGCTTATTACAGTGGCAA Reverse: GTCGGAGATTCGTAGCTGGA

### 2.4 Celastrol inhibits NF-κB and AKT1 phosphorylation in high glucose-induced senescent renal tubular epithelial cells

**FIGURE 4:**
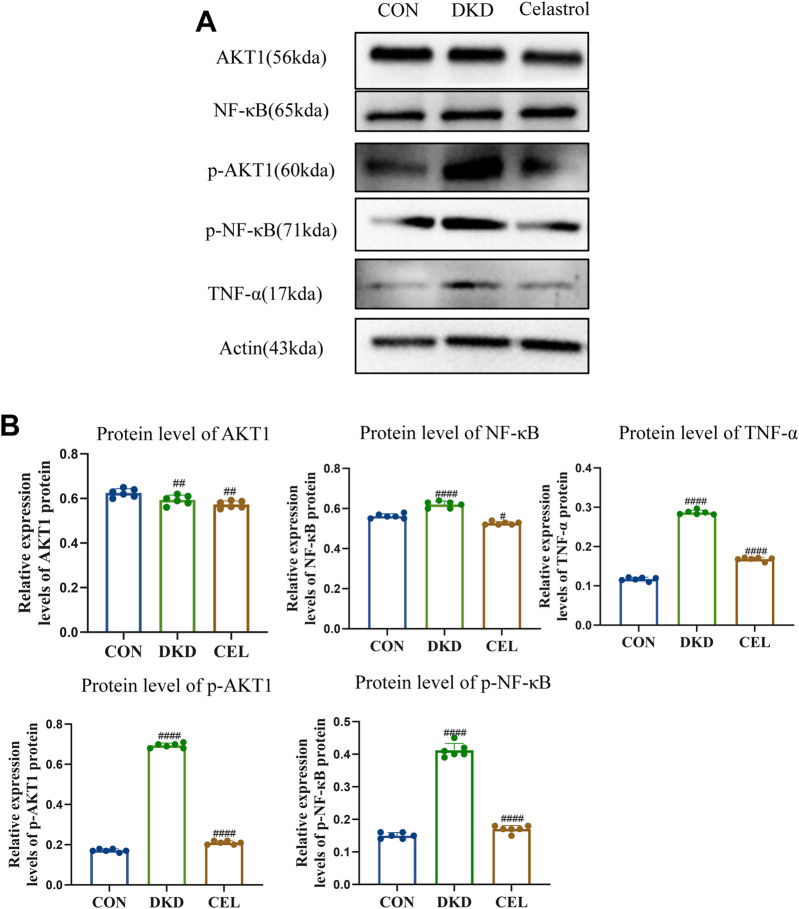
Celastrol Inhibits NF-κB and AKT1 Phosphorylation. **(A)** Western blot results following treatment of celastrol *in vitro*. Among them, ET stands for exposure time. **(B)** Statistical results of Western blot analysis following treatment of celastrol (n = 6, all data are presented as mean ± SD, *p < 0.05, **p < 0.01, ***p < 0.001; #p < 0.05, ##p < 0.01, ###p < 0.001,compared with the control group; *p < 0.05, **p < 0.01, ***p < 0.001; #p < 0.05, ##p < 0.01, ###p < 0.001,compared with the DKD group).

NF-κB serves as a pivotal regulatory factor in inflammatory responses and a critical therapeutic target for inflammatory diseases, while the PI3K/Akt signaling pathway plays a significant role in promoting the progression of diabetic kidney disease (DKD) (Zhang et al., 2019; En et al., 2021). Western blotting (WB) analysis revealed that celastrol effectively inhibits the intracellular phosphorylation levels of p-NF-κB and p-AKT1 proteins (Figures 4A,B). Furthermore, TNF-α, a key mediator in initiating innate immune inflammatory responses—induced by bacterial pathogens and harmful stimuli through Toll-like receptors and NF-κB signaling—has been reported to promote extracellular matrix (ECM) marker secretion (e.g., type I collagen and fibronectin) in proximal tubular epithelial cells, accelerating renal tubulointerstitial fibrosis (Reidy et al., 2014; Akira and Takeda, 2004).

Experimental results demonstrated that high-glucose conditions upregulated TNF-α expression, which was significantly attenuated by celastrol treatment, underscoring the compound’s anti-inflammatory potential. Mechanistically, Tripterygium wilfordii, a source of celastrol, likely exerts its therapeutic effects—including anti-inflammatory and antioxidative activities—by suppressing the phosphorylation of NF-κB and AKT1 signaling pathways, thereby mitigating pathological processes linked to inflammation and DKD progression.

### 2.5 Celastrol improves renal function in DKD rats by reducing senescent cells

**FIGURE 5:**
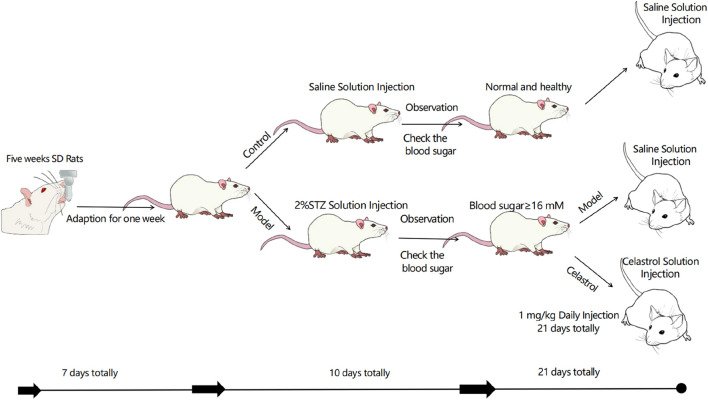
*In vivo* experimental procedure.

**FIGURE 6:**
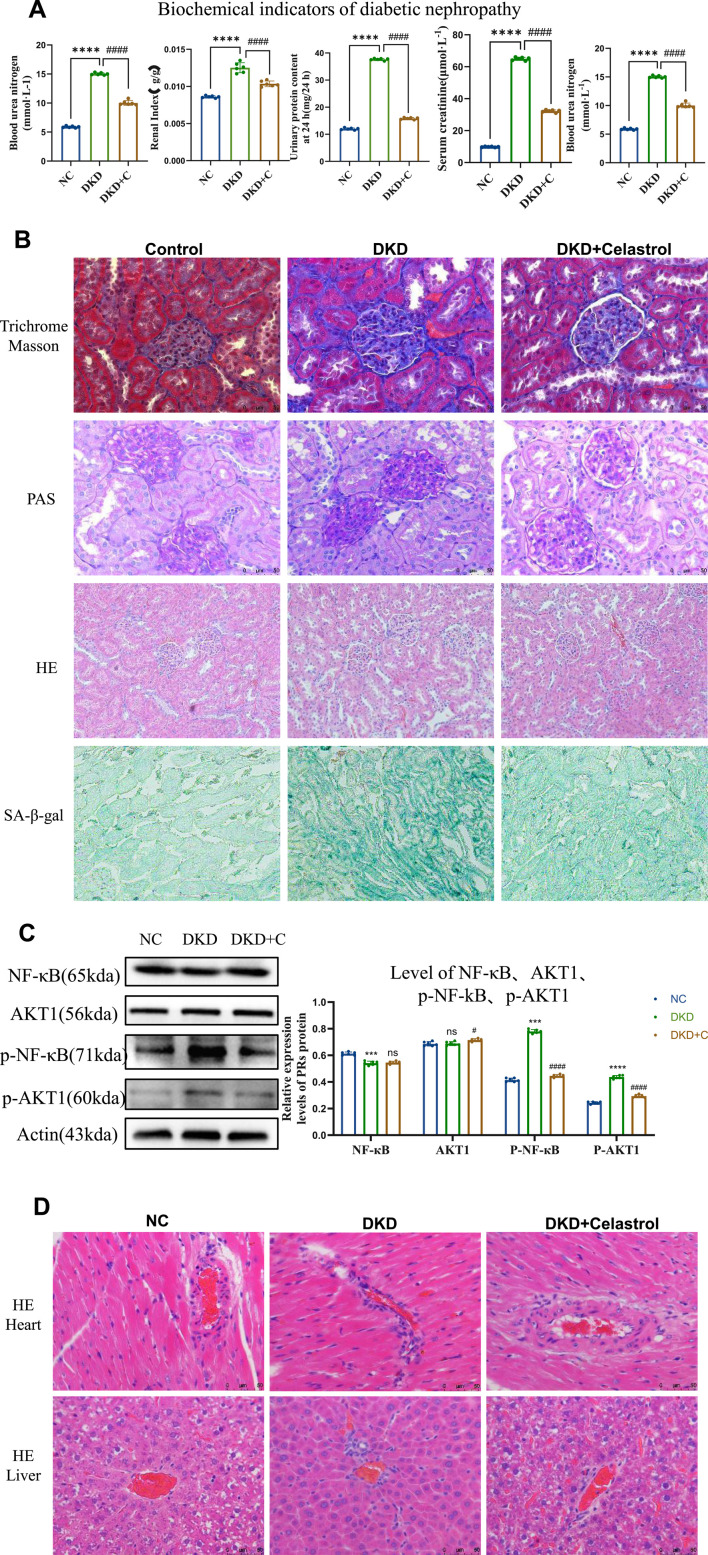
Celastrol Improves Renal Function in DKD Rats. **(A)** Comparison of blood glucose levels, renal coefficient, 24-h urinary protein content, serum creatinine, and serum urea nitrogen among groups following administration of celastrol (n = 6, all data are presented as mean ± SD, *p < 0.05, **p < 0.01, ***p < 0.001; #p < 0.05, ##p < 0.01, ###p < 0.001,compared with the control group; *p < 0.05, **p < 0.01, ***p < 0.001; #p < 0.05, ##p < 0.01, ###p < 0.001,compared with the DKD group). **(B)** Effects of celastrol on histopathological changes in the kidneys of diabetic nephropathy (H&E staining images of the NC group, DKD group, and DKD + C group (200×); PAS staining images (400×); MASSON staining images (400×); β-galactosidase staining images (200×)). **(C)** Statistical results of histone immunostaining among groups following administration of celastrol (n = 6, all data are presented as mean ± SD,*p < 0.05, **p < 0.01, ***p < 0.001; #p < 0.05, ##p < 0.01, ###p < 0.001,compared with the control group; *p < 0.05, **p < 0.01, ***p < 0.001; #p < 0.05, ##p < 0.01, ###p < 0.001,compared with the DKD group). **(D)** H&E staining images of tissue sections from each group following administration of celastrol (400×).

*In vivo* experiments validated celastrol’s therapeutic efficacy in streptozotocin (STZ)-induced diabetic kidney disease (DKD) rats. DKD rats exhibited weight loss, polyuria, hyperglycemia, and elevated 24-h urinary protein, serum creatinine, and urea nitrogen levels compared to normal controls. This is a schematic diagram of our *in vivo* animal experiment(Figure 5). Celastrol treatment significantly reduced hyperglycemia in DKD rats (DKD + C group), with no effect in normal (NC) or untreated DKD groups. Renal injury was assessed via kidney index (kidney weight/body weight ratio), which was elevated in DKD rats but normalized in DKD + C rats (Figure 6A). Biochemical analyses further revealed that celastrol reduced 24-h urinary protein, serum creatinine, and urea nitrogen levels in DKD + C rats (Figure 6A).

Histopathological evaluation showed glomerular lobulation, vascular atrophy, cytoplasmic vacuolization, mesangial matrix expansion (PAS staining), interstitial fibrosis (Masson staining), and senescence (β-galactosidase staining) in DKD kidneys. These abnormalities were significantly attenuated in DKD + C rats, indicating improved renal histopathology. Mechanistically, celastrol suppressed phosphorylated NF-κB and AKT1 expression in renal tissues (Figures 6B,C), consistent with *in vitro* findings.

Notably, histological analysis of heart and liver tissues revealed no significant pathological changes after celastrol treatment (Figure 6D), demonstrating no cardiotoxicity or hepatotoxicity at the tested dose. Collectively, these results confirm that celastrol ameliorates DKD pathology *in vivo* by inhibiting NF-κB and AKT1 phosphorylation without overt systemic toxicity.

## 3 Materials and methods

### 3.1 Culturing of human cells

Human renal tubular epithelial cells (HK-2) were purchased from Wuhan Prosel Life Science and Technology Co., Ltd. The cell line was authenticated by short tandem repeat (STR) identification, as reported by the supplier. HK-2 cells were cultured in a basal sugar-free medium (DMEM, #PM150270, Procell, Wuhan) supplemented with 1% penicillin-streptomycin solution (PLS) and 10% fetal bovine serum (FBS) in a humidified incubator at 37 °C with 5% CO_2_, The cells used were at passages 1 to 9. When the cell confluence reached 80%–90%, the cells were trypsinized. After reattachment, the medium was replaced with one containing 0.5% serum and cultured for 24 h to maintain the cells in a resting state. The HK-2 cells were divided into the following groups: (1) control group (CON, glucose 0 mmol/L), (2) high glucose group (DKD, glucose 30 mmol/L), (3) celastrol low-dose group (L, 50 nmol/L), (4) celastrol medium-dose group (M, 250 nmol/L), (5) celastrol high-dose group (H, 500 nmol/L). All treatment groups were cultured in high-glucose medium (glucose 30 mmol/L) for 48 h, after which the supernatant and cells were collected for further analysis.

### 3.2 Animal models of diabetic kidney disease

*In vivo* experiments were conducted using male Sprague-Dawley (SD) rats (n = 30, body weight 140–150 g) purchased from Beijing Huafukang Biotechnology Co., Ltd. Animal works had been approved by the reserach ethics committe of School of Pharmaceutical Science, Shandong First Medical University. The rats were housed individually in ventilated cages under specific-pathogen-free (SPF) conditions in a controlled environment (temperature 20 °C–24 °C, relative humidity 30%–70%, positive pressure) with free access to food and water. The breeding environment was maintained with a pressure difference of (14 ± 1) Pa, and the bedding was changed daily to ensure dryness.

After a 1-week adaptation period, the rats were randomly divided into a normal control group (n = 10) and a model group (n = 20). The model group rats were intraperitoneally injected with 50 mg/kg streptozotocin (STZ, freshly dissolved in precooled citrate buffer solution, pH 4.5, #BS185-1g, Biosharp, Anhui, China) twice a week for 3 weeks. One week after the initial STZ injection, rats with blood glucose levels >16.6 mmol/L were selected and further divided into the DKD group (n = 8) and the tripterine group (DKD + C, 1 mg/kg, n = 8). The normal control group (NC) rats received intraperitoneal injections of STZ solvent (precooled citrate buffer, pH 4.5) for 3 weeks. The mortality rate of the DKD rat model was approximately 20%. At the end of the experiment, the rats were anesthetized with sodium pentobarbital, bled from the abdominal aorta, and euthanized by cervical dislocation. Tissue samples were collected for further analysis.

### 3.3 Cell viability assay

Cell viability was measured using the CCK-8 assay. HK-2 cells were seeded in 96-well plates at a density of 8 × 10^3^ cells per well and cultured overnight to allow adherence. Subsequently, the cells were treated with glucose-containing medium of different concentrations for 24 h (20, 50, 30, 100, 200 mM). This experiment is used to screen for the appropriate concentration that induces cellular senescence. After the treatment, the CCK-8 reagent was diluted with medium at a ratio of 1:10 (resulting in a final concentration of 10%), added to the wells, and the cells were further cultured for 4 h. Absorbance values were measured at a wavelength of 450 nm using a multimode microplate reader (SpectraMax® M2). After exporting and processing the data, the half-maximal inhibitory concentration (IC50) was calculated.

Cell viability was assessed using the CCK-8 assay. HK-2 cells were seeded in 96-well plates at a density of 8 × 10^3^ cells per well and allowed to attach overnight. The cells were then exposed to different concentrations of celastrol and glucose-containing medium for 24 h. This experiment is used to screen for the appropriate concentration of celastrol to be administered. After treatment, the CCK-8 reagent was diluted 1:10 with the medium to a final concentration of 10%, and the cells were cultured for an additional 4 h. Absorbance was measured at a wavelength of 450 nm using a multimode microplate reader (SpectraMax® M2). Data were exported and processed, and the IC_50_ value was calculated.

### 3.4 Senescence induction and SA-β-gal staining

For senescence induction, cells were cultured in the presence of 30 mM glucose in DMEM containing 10% FBS and 1% P/S(Penicillin-Streptomycin) for 24 h. Cells were seeded in 24-well plates at a density of 1 × 10^4^ cells per well. Senescence-associated β-galactosidase (SA-β-Gal) staining was performed by fixing the cells in SA-β-Gal fixation solution (PBS with 0.1 M EGTA, 1 M MgCl_2_, and 50% glutaraldehyde) for 15 min at room temperature, followed by washing three times with PBS. A staining solution at pH 6 (PBS with 1 M MgCl_2_, 0.5 M K_4_ (Fe (CN)_6_), 0.5 M K_3_ (Fe (CN)_6_), and 1 mg/mL X-GAL diluted in dimethylformamide) was added, and the cells were incubated overnight at 37 °C in a CO_2_-free incubator.

### 3.5 Measurement of ROS

Reactive oxygen species (ROS) were measured using 2′,7′-dichlorodihydrofluorescein diacetate (DCFH-DA). Cells were seeded in 24-well plates at a density of 8 × 10^3^ cells per well and cultured overnight. They were then treated with 30 mM glucose for 24 h, followed by re-treatment with different concentrations of celastrol (50, 250, 500 nM). A control group without treatment was also established. The cells were washed with serum-free medium and incubated with DCFH-DA at 37 °C in the dark for 20 min. DCFH-DA is deacetylated by intracellular esterases to a non-fluorescent compound, which is then oxidized by ROS to fluorescent 2′,7′-dichlorofluorescein (DCF). Cell nuclei were labeled with Hoechst 33,342, and DCF fluorescence was detected using a fluorescence microscope and a microplate reader (excitation wavelength 488 nm, emission wavelength 525 nm).

### 3.6 Measurement of JC-1

Mitochondrial membrane potential was assessed using the JC-1 fluorescent probe. Cells were seeded in 24-well plates at a density of 8 × 10^3^ cells per well and cultured overnight. They were then treated with 30 mM glucose for 24 h, followed by re-treatment with different concentrations of celastrol (50, 250, 500 nM). A control group without treatment was also established. The cells were washed with PBS and incubated with JC-1 at 37 °C in the dark for 20 min. JC-1 aggregates in the mitochondrial matrix to form polymers (J-aggregates) that emit red fluorescence. When the mitochondrial membrane potential is low, JC-1 exists as a monomer and emits green fluorescence. The ratio of red to green fluorescence was used to determine the extent of mitochondrial depolarization. Cell nuclei were labeled with Hoechst 33,342, and detection was performed using a fluorescence microscope or a microplate reader.

### 3.7 Western blotting

Cells treated with celastrol or 30 mM glucose were lysed in 1× sodium dodecyl sulfate (SDS) loading buffer. The lysates were separated by SDS-polyacrylamide gel electrophoresis (SDS-PAGE) and transferred onto a nitrocellulose membrane (Bio-Rad Laboratories, Richmond, CA). The membrane was blocked with bovine serum albumin (BSA) and incubated with primary antibodies against the target proteins overnight. Secondary antibodies (Proteintech) were applied on a shaker at room temperature for 1 h. Detection was performed using an Ultra-sensitive ECL Chemiluminescence Detection Kit (Proteintech Biotechnology Co. Ltd., China).

### 3.8 Real-time quantitative PCR

Total RNA was extracted from cell samples using the Cell/Tissue Total RNA Extraction Kit (Yeasen Biotechnology Co., Ltd., China) according to the manufacturer’s instructions. RNA concentration and purity were measured using a Nanodrop micro-spectrophotometer. Reverse transcription of cDNA was performed using the 4×Hifair® Ⅲ SuperMix plus system. The cDNA was amplified using SYBR Green PCR premix (Yeasen Biotechnology Co., Ltd., China). The primers used for quantitative real-time polymerase chain reaction are listed in the Supplementary Table.

### 3.9 Animal treatment

Male SD rats (5 weeks old, body weight 150 ± 5 g) were purchased from Beijing Huafukang Biotechnology Co., Ltd. The diabetic kidney disease model was established by injecting rats with STZ solution at a dose of 50 mg/kg based on body weight. The injection volume was calculated by dividing the required mass of STZ by 2%. The rats were divided into two groups based on blood glucose and 24-h urinary protein content. Each group of rats received intraperitoneal injections of a solution (1% DMSO) and 1 mg/kg of celastrol (daily for 21 days). After 3 weeks, the rats were sacrificed, and various organs were collected. The animal experiment was conducted in accordance with all ethical regulations related to animal research and was approved by the Laboratory Animal Management and Use Committee of the School of Pharmacy, Shandong First Medical University.

### 3.10 Detection of biochemical indicators

Blood and urine samples were collected from rats in the control group (injected with normal saline), the model group (injected with STZ), and the celastrol group (injected with celastrol). Blood samples were used to detect fasting blood glucose (using a blood glucose meter) and to measure serum creatinine and blood urea nitrogen levels. Blood samples taken from the rat eyeballs were allowed to clot at room temperature for 30–60 min, then centrifuged at 4 °C for 10–15 min. The supernatant was collected for subsequent experiments. Urine samples were used to detect 24-h urinary protein content using the bicinchoninic acid method.

### 3.11 Histopathological evaluation

Tissue-level pathological changes of diabetic kidney disease in rats were detected using Masson’s trichrome staining and hematoxylin-eosin (H&E) staining. Glycogen deposition in rat kidney tissues was detected using periodic acid-Schiff (PAS) staining.

### 3.12 Statistical analyses

Image analysis was performed using ImageJ software (version 1.48). Data from at least six groups of samples were pooled for statistical analysis, with at least three biological replicates and two technical replicates. Statistical analysis was performed using GraphPad Prism 9.0 (GraphPad Software, Inc., La Jolla, CA, United States of America). Prism software (GraphPad) was used to plot graphs and the results were given as the mean ± standard deviation (SD). Comparisons between two groups were analyzed using an unpaired two-tailed Student’s t-test. For comparisons among multiple groups, one-way analysis of variance (ANOVA) followed by Tukey’s *post hoc* test was used. A p-value of less than 0.05 was considered statistically significant. (*p < 0.05, **p < 0.01, ***p < 0.001; #p < 0.05, ##p < 0.01, ###p < 0.001).

## 4 Discussion

The detrimental role of cellular senescence in the pathogenesis and progression of Diabetic Nephropathy (DN) or Diabetic Kidney Disease (DKD) is increasingly recognized. Research indicates that renal cells, including tubular epithelial cells, mesangial cells, and podocytes, undergo premature senescence in the diabetic milieu due to factors like hyperglycemia, oxidative stress, and the accumulation of advanced glycation end-products (AGEs) (Shen et al., 2022). The accumulation of these senescent cells in the kidney is particularly concerning because they develop a senescence-associated secretory phenotype (SASP), characterized by the release of a potent cocktail of pro-inflammatory cytokines, chemokines, growth factors, and extracellular matrix remodeling enzymes (Li et al., 2023; G et al., 2022; Li and Lerman, 2020; Wei et al., 2024).

One fundamental mechanism is DNA damage, often triggered by hyperglycemia-induced oxidative stress and the accumulation of advanced glycation end-products (AGEs). This, in turn, leads to the activation of the p53-p21-Rb pathway, a key cell cycle arrest pathway, ultimately halting cell proliferation and promoting senescence. Similarly, telomere attrition, accelerated by inflammatory and metabolic stressors in diabetes, also activates the p53-p21 pathway, contributing to replicative senescence (Liu J. et al., 2024). Mitochondrial dysfunction is another central player. Diabetic conditions lead to mitochondrial damage, increased production of reactive oxygen species (ROS), and impaired energy metabolism. Dysfunctional mitochondria can directly trigger senescence, and their inability to efficiently clear ROS further exacerbates cellular damage, creating a vicious cycle (Takasu et al., 2025; Miwa et al., 2022; Hoogstraten et al., 2024). The involvement of cellular senescence in Diabetic Nephropathy (DN) or Diabetic Kidney Disease (DKD) is a complex process driven by several interconnected mechanisms and pathways within various renal cell types.

Mitochondrial dysfunction is another central player. Diabetic conditions lead to mitochondrial damage, increased production of reactive oxygen species (ROS), and impaired energy metabolism. Dysfunctional mitochondria can directly trigger senescence, and their inability to efficiently clear ROS further exacerbates cellular damage, creating a vicious cycle (Wei et al., 2024).

The accumulation of these senescent renal cells, regardless of the specific initiating mechanism, culminates in the release of the senescence-associated secretory phenotype (SASP) (Cuollo et al., 2020; Huang et al., 2022). This complex secretome, rich in pro-inflammatory cytokines (e.g., IL-6, TNF-α), chemokines, growth factors (e.g., TGF-β), and matrix metalloproteinases, creates a detrimental microenvironment. The SASP not only perpetuates inflammation and promotes renal fibrosis but also impairs the regenerative capacity of the kidney, accelerating the decline in renal function characteristic of DKD (Wei et al., 2024). Understanding these intricate mechanisms and pathways is essential for developing novel and targeted therapeutic strategies to combat DN/DKD.

Based on differential expression immune genes (DEIG), weighted gene co-expression network, and protein-protein network analyses, Chen et al. identified CCL19 as a central immune-related biomarker (Chen et al., 2022). Moreover, there is increasing evidence that the excessive production of reactive oxygen species (ROS) is the common point between the alteration of renal metabolic pathways and the disruption of renal hemodynamics related to diabetic kidney disease (DKD). These changes ultimately lead to the occurrence of inflammation, fibrosis, and endothelial dysfunction. Generally, renal oxidative stress is caused by the upregulation of ROS production induced by pro-oxidant enzymes and the subsequent consumption of antioxidants. Excessive ROS production will not only result in the occurrence of renal fibrosis and inflammation but also trigger severe tissue damage through processes such as promoting lipid peroxidation, DNA damage, protein modification, and mitochondrial dysfunction. Therefore, in-depth exploration of the mechanism of action of ROS in kidney diseases is of great significance for understanding the pathogenesis of DKD and developing effective treatment strategies (Jha et al., 2016).

Furthermore, mitochondria are dynamic organelles that undergo frequent fission and fusion events regulated by fission proteins and fusion proteins, maintaining mitochondrial turnover and the balance of the cellular network (Xiao et al., 2014). Dysfunctional mitochondria exhibit fragmentation and membrane depolarization, generate large amounts of reactive oxygen species and release apoptotic proteins in response to stressors such as diabetic kidney disease (DKD), and ultimately activate the mitochondrial cell death pathway. Renal proximal tubular cells contain abundant mitochondria and rely on oxidative phosphorylation. Therefore, tubular cells are prone to mitochondrial damage (Xiao et al., 2017). Excessive mitochondrial oxidative stress and abnormal dynamics are the main factors contributing to tubular injury in DKD (Zhan et al., 2015; Chien et al., 2011; Shen et al., 2022; Ma et al., 2022).

Therefore, in this study, the expression level of CCL19 in cells, the level of reactive oxygen species (ROS), and the degree of mitochondrial damage were taken as important indicators for the establishment of the *in vitro* model of diabetic kidney disease (DKD) and the screening of components of Tripterygium wilfordii. The experimental results also showed that a glucose concentration of 30 mM was the optimal concentration for inducing the *in vitro* model of DKD. Under this concentration condition, the expression level of CCL19 (a biomarker of DKD in renal tubular epithelial cells), the intracellular ROS level, and the degree of mitochondrial damage were all significantly increased.

Recent research findings have revealed that diabetic kidney disease (DKD) is closely related to the accelerated senescence of various cells such as renal tubular epithelial cells, podocytes, mesangial cells, and endothelial cells. Notably, hyperglycemia can directly induce the senescence of mesangial cells and renal tubular epithelial cells. In addition, the activity of SA-β-Gal (senescence-associated β-galactosidase) and the overexpression of p16INK4A in renal tubular epithelial cells are positively correlated with renal interstitial fibrosis and tubular cell atrophy. It is worth noting that the senescence of renal tubular cells is closely related to body mass index and blood glucose levels, which means that controlling cell senescence plays a crucial role in the treatment of DKD. The key to senescence-associated secretory phenotype (SASP) transcription lies in the phosphorylation of the transcription factors NF-κB and AKT1. Studies have reported that the phosphorylated NF-κB p65/RelA subunit translocates to the nucleus, where it binds to the promoters of several SASP genes, thereby promoting the induction and regulation of cell senescence or DNA damage (Li and Lerman, 2020). Meanwhile, renal tubular epithelial cells are particularly vulnerable to the disorders in the diabetic state because of their high energy requirements and dependence on aerobic metabolism. Hyperglycemia, oxidative stress, persistent chronic inflammation, glucose toxicity, accumulation of advanced glycation end products (AGEs), lipid metabolism disorders, and lipotoxicity lead to the senescence of renal tubular epithelial cells and different forms of regulated cell death. Experimental results showed that senescent renal tubular epithelial cells accounted for about 80% under the condition of a 30 mM glucose concentration, and the levels of intracellular p-NF-κB and p-AKT1 were significantly increased after treatment with 30 mM glucose.

Based on the successful establishment of the above *in vitro* model of diabetic kidney disease (DKD), ROS (reactive oxygen species) activity detection and JC-1 staining experiments were then carried out on celastrol. Through statistical analysis, it was finally found that celastrol had a significant effect. Celastrol is a pentacyclic triterpenoid compound isolated from the roots of Tripterygium wilfordii and is also one of the main active ingredients of Tripterygium wilfordii.

Then we conducted research on the mechanism of action of Tripterygium wilfordii. Through literature review, it was found that Huobahua Gen Pian (CRT), which is processed from the roots of Tripterygium hypoglaucum (Lévl.) Hutchins belonging to the genus Tripterygium in the Celastraceae family, was studied by Zhaochen Ma et al. According to network analysis, the main network targets of CRT in anti-diabetic kidney disease (DKD) were significantly enriched in the PI3K/AKT/NF-κB pathway, and this pathway may play a central role in regulating the inflammatory responses of various diseases. Through immunoblotting analysis, they found that compared with the normal control group, the expression levels of p-PI3K, p-AKT, and p-NF-κB proteins in the kidney tissues of DKD rats and HK-2 cells under high-glucose stimulation were all significantly increased. Meanwhile, the ratios of p-PI3K to total PI3K, p-AKT to total AKT, and p-NF-κB to total NF-κB also showed a significant upward trend. These results further supported the important role of the PI3K/AKT/NF-κB pathway in the process of CRT against DKD. Therefore, in this study, the systems of NF-κB, AKT1, as well as p-NF-κB and p-AKT1 were also selected for research, and the obtained results were consistent with those in the above literature. It was proved that celastrol inhibited the phosphorylation of NF-κB and AKT1 *in vitro*. Moreover, the expression level of the downstream protein TNF-α was consistent with that of the upstream, which was in line with the experimental expectations.

It can be known through literature review that for the establishment of the diabetic kidney disease (DKD) model, Sprague-Dawley (SD) rats are usually used, and the modeling method is quite mature and commonly adopted. In this study, the streptozotocin (STZ) injection method was employed. After modeling, the success of the model was judged by detecting blood glucose, body weight, urinary protein, serum creatinine, serum urea nitrogen and the condition of the rats. Due to the relatively high mortality rate caused by feeding with a high-sugar and high-fat diet combined with STZ injection, only the STZ injection method was adopted. In this way, the rats developed type 1 diabetes and would inevitably have complications of DKD as well. However, due to individual differences among the rats after modeling, although the blood glucose levels of the successfully modeled rats were all higher than 16 mM, some rats had blood glucose levels lower than 20 mM, while some could be as high as 30.9 mM.

Based on the rat disease model of DKD, in order to further study the mechanism of action of celastrol, a preliminary experiment was carried out before the formal start of the animal experiment in this study to determine the dosage of celastrol *in vivo*. Three concentrations, namely, low, medium and high, were used for drug administration in this study. Due to the relatively strong toxicity of celastrol, rats in the medium-dose (2 mg/kg) and high-dose (3 mg/kg) groups had adverse reactions such as black secretions at the corners of the eyes and on the nose, ulceration and scabbing of the abdominal skin, and abdominal adhesion. Therefore, the low dose (1 mg/kg) was selected for daily administration in this study. The effect was not obvious in the first week, and the effect of drug administration began to appear in the second week. In this experiment, the blood glucose and body weight of the rats were detected every 5 days, and their conditions were observed.

In conclusion, celastrol can not only reduce the level of reactive oxygen species in cells but also alleviate mitochondrial damage. This study also found that celastrol can inhibit the expression of proteins that accelerate cell senescence and reverse cell senescence caused by high glucose. In addition, celastrol can decrease the expression levels of senescence-associated secretory phenotype (SASP) factors and senescence-related markers in diabetic kidney disease (DKD) cells. *In vivo* experiments also demonstrated that it can improve the pathological changes in kidney tissues. Moreover, compared with some other components, celastrol requires a much lower drug concentration, which provides a basis for rational drug use. The use of a small dosage will also reduce the metabolic burden on patients. Thus, celastrol exhibits significant therapeutic potential for DKD by modulating the AKT/NF-κB/TNF-α signaling pathways and mitigating cellular senescence. Its ability to improve renal function and pathology in both cellular and animal models highlights its promise as a candidate for DKD treatment.

## Data Availability

The datasets presented in this study can be found in online repositories. The names of the repository/repositories and accession number(s) can be found in the article/Supplementary Material.
