# Supplementary material for: Celastrol attenuates diabetic kidney disease progression by repressing senescence of renal tubular epithelial cells
Source: Front Aging. 2025 Nov 27;6:1657947. doi: 10.3389/fragi.2025.1657947 (PMC12696166; doi:10.3389/fragi.2025.1657947)
Supplement: Supplementary file 1 [file Presentation1.pptx]

## Slide 1
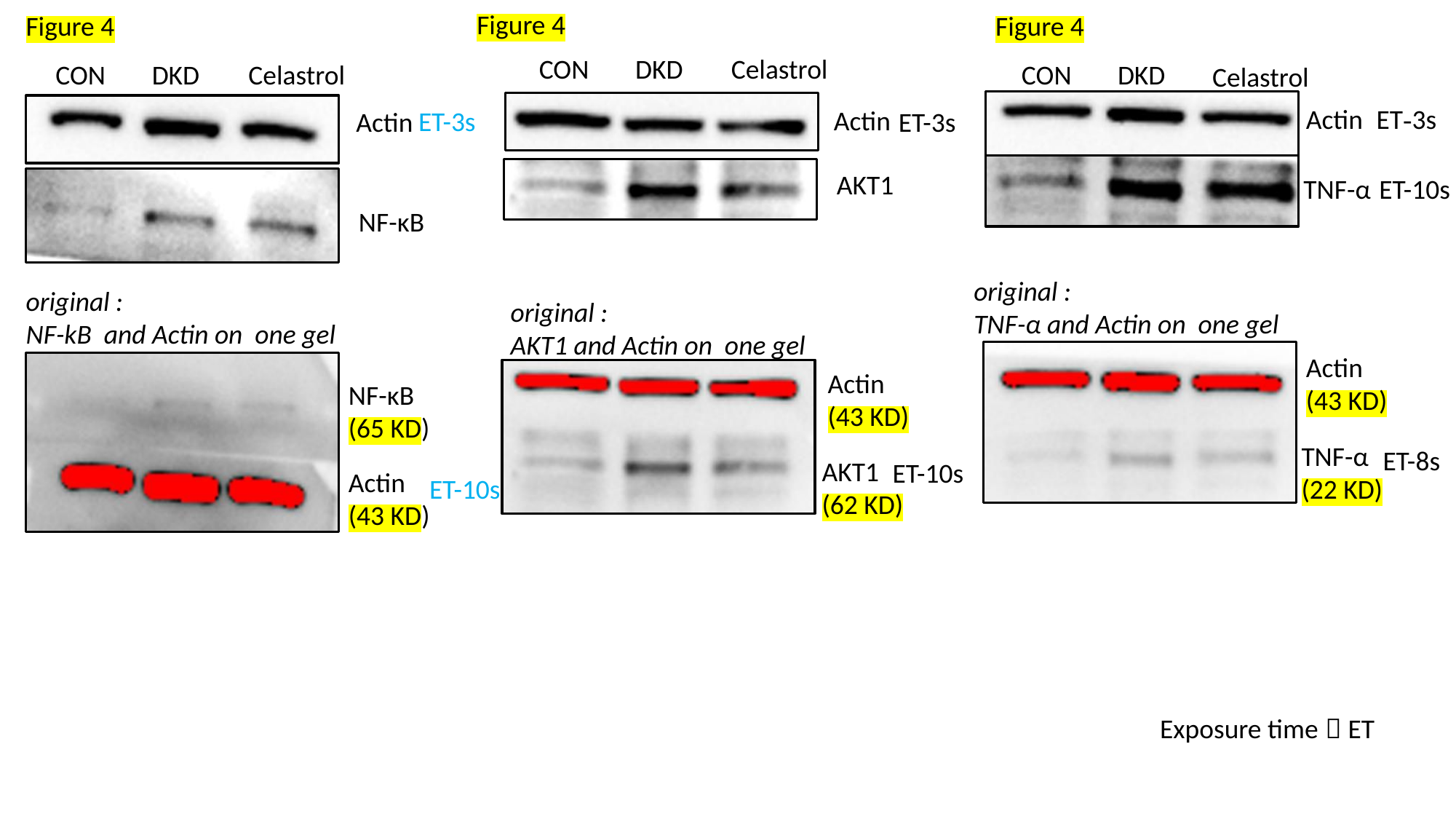

Figure 4
Figure 4
Figure 4
Celastrol
CON
DKD
CON
DKD
Celastrol
ET-3s
Actin
NF-κB
NF-κB
(65 KD)
Actin
(43 KD)
ET-10s
CON
DKD
Celastrol
ET-3s
Actin
TNF-α
ET-10s
Actin
(43 KD)
TNF-α
(22 KD)
ET-8s
Actin
ET-3s
AKT1
original :
TNF-α and Actin on one gel
original :
NF-kB and Actin on one gel
original :
AKT1 and Actin on one gel
Actin
(43 KD)
ET-10s
AKT1
(62 KD)
Exposure time：ET

## Slide 2
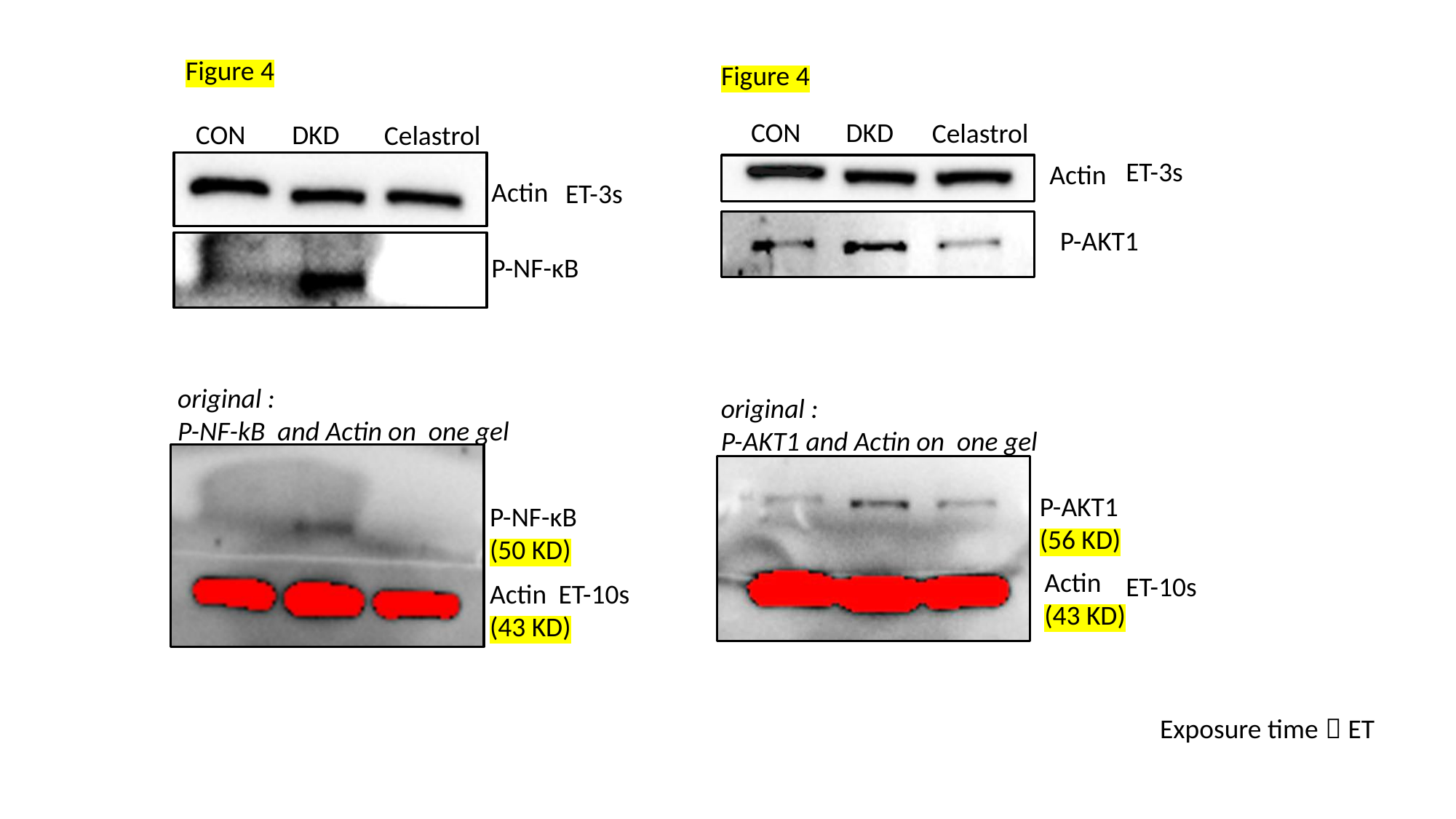

Figure 4
Figure 4
CON
DKD
Celastrol
CON
DKD
Celastrol
ET-3s
Actin
Actin
ET-3s
P-AKT1
P-NF-κB
original :
P-NF-kB and Actin on one gel
P-NF-κB
(50 KD)
Actin
(43 KD)
ET-10s
original :
P-AKT1 and Actin on one gel
P-AKT1
(56 KD)
Actin
(43 KD)
ET-10s
Exposure time：ET
